# Effects of tetrahedral framework nucleic acid/wogonin complexes on osteoarthritis

**DOI:** 10.1038/s41413-019-0077-4

**Published:** 2020-02-10

**Authors:** Shi Sirong, Chen Yang, Tian Taoran, Li Songhang, Lin Shiyu, Zhang Yuxin, Shao Xiaoru, Zhang Tao, Lin Yunfeng, Cai Xiaoxiao

**Affiliations:** 1 State Key Laboratory of Oral Disease, National Clinical Research Center for Oral Diseases, West China Hosptial of Stomatology, Sichuan University, Chengdu, 610041 China; 2 Department of Liver Surgery & Liver Transplantation Center, West China Hospital of Sichuan University, Chengdu, 610041 Sichuan Province China

**Keywords:** Bone, Bone quality and biomechanics

## Abstract

Osteoarthritis, a disorder characterized by articular cartilage deterioration, varying degrees of inflammation, and chondrocyte apoptosis, is the most common chronic joint disease. To slow or reverse its progression, inflammation should be inhibited, and chondrocyte proliferation should be promoted. Tetrahedral framework nucleic acids can be internalized by chondrocytes (even inflammatory chondrocytes) and can enhance their proliferation and migration. Wogonin, a naturally occurring flavonoid, suppresses oxidative stress and inhibits inflammation. In this study, tetrahedral framework nucleic acids were successfully self-assembled and used to load wogonin. We confirmed the effective formation of tetrahedral framework nucleic acid/wogonin complexes by dynamic light scattering, zeta potential analysis, transmission electron microscopy, and fluorescence spectrophotometry. Tetrahedral framework nucleic acids, wogonin, and especially tetrahedral framework nucleic acid/wogonin complexes effectively alleviated inflammation in vitro and in vivo and prevented cartilage destruction. In addition, these materials remarkably downregulated the expression of inflammatory mediators and matrix metalloproteinases, upregulated chondrogenic markers, and promoted tissue inhibitor of metalloproteinase 1 and B-cell lymphoma 2 expression. In vivo, after treatment with tetrahedral framework nucleic acid/wogonin complexes, the bone mineral density in regenerated tissues was much higher than that found in the untreated groups. Histologically, the complexes enhanced new tissue regeneration, significantly suppressed chondrocyte apoptosis, and promoted chondrogenic marker expression. They also inhibited cell apoptosis, increased chondrogenic marker expression, and suppressed the expression of inflammatory mediators in osteoarthritis. Therefore, we believe that tetrahedral framework nucleic acid/wogonin complexes can be used as an injectable form of therapy for osteoarthritis.

## Introduction

Osteoarthritis (OA), which is characterized by osteophyte formation, slight synovial inflammation, subchondral bone lesions, and destruction of articular cartilage, is the most common chronic joint disease.^1–3^ The incidence of OA has been demonstrated to increase with age, especially in patients over the age of 65 years.^4–6^ It is estimated that more than 100 million people are affected by OA worldwide.^7^ These individuals experience pain, swelling, stiffness, joint deformation and dysfunction, limited motion, and functional disability throughout their daily lives. Current treatment options for OA, namely, conventional nonsteroidal anti-inflammatory drugs and hyaluronic acid, mainly act by inhibiting inflammation and reducing pain. In developed countries, total knee arthroplasty is currently one of the most effective treatments for OA. Treating OA is associated with high costs,^8^ and its occurrence and progression are associated with various risk factors, such as aging, genetics, obesity, overuse, infection, and joint injury.^9,10^ Cartilage is well known as an avascular connective tissue in which the sole cell type, the chondrocyte, obtains nutrition, and oxygen via diffusion from synovial fluid and subchondral bone.^11–13^ Substantial evidence has revealed that chondrocyte apoptosis, chondrocyte phenotype loss, and synovial inflammation, which are characterized by the overproduction of pro-inflammatory mediators (including tumor necrosis factor-α (TNF-α) and interleukin (IL)-1β) play a vital role in the occurrence and progression of OA, particularly in the early stages.^14–16^ Multiple signaling pathways and cytokines are involved in the apoptosis of chondrocytes in OA. Due to the high levels of pro-inflammatory cytokines and the many dead chondrocytes in OA joints, recent research of OA treatment has focused on exploring agents that can inhibit chondrocyte apoptosis, promote chondrocyte proliferation, and reduce the levels of catabolic factors involved in OA to slow or reverse OA progression.^17–20^

In terms of chondroprotective effect, we found that tetrahedral framework nucleic acid (TFNA), a specific, novel, and very promising DNA nanomaterial, can maintain the morphology of chondrocytes and enhance chondrocyte proliferation by activating the Notch signaling pathway and promote the migration of chondrocytes by activating the RhoA/ROCK signaling pathway at an optimum concentration of 250 nmol·L^−1^.^21–23^ TFNA can also be easily synthesized using several unique methods and specifically designed single-stranded DNA (ssDNA) by utilizing unique and sophisticated Watson–Crick base pairing.^24^ Accumulating evidence has shown that TFNA but not ssDNA or other DNAs can permeate cells and enter mammalian cells via caveolin-mediated endocytosis, in which it remains intact for up to 48 h. Meanwhile, TFNA can escape from lysosomes via nuclear localization signals, which play a considerable and vital role in gene delivery.^25,26^ Due to its mechanical rigidity, structural stability, and efficient functionalization, TFNA has been explored and functionalized using DNA fragments, fluorophores, various RNAs, single-molecule proteins, and small molecular drugs such as siRNAs, microRNAs, CpGs, and metal complexes.^27–33^

Wogonin (5,7-dihydroxy-8-methoxyflavanone), a natural flavonoid and a traditional Chinese medicine, is isolated from the root extract of *Scutellaria baicalensis*. Wogonin exerts many biological activities, such as anti-inflammatory and anti-cancer activities,^34,35^ and importantly, it does not exhibit significant toxicity in normal tissues.^36^ Wogonin has also been shown to suppress key mediators of oxidative stress, inhibit inflammation, and matrix degradation via suppression of major proteases, including matrix metalloproteinase (MMP)-3, MMP-9, and MMP-13, and promote the expression of chondrogenic markers such as COL2A1 and aggrecan (AGC) in OA chondrocytes and cartilage explants.^37–39^ Above all, wogonin can bind to DNA via incorporation as a DNA intercalator and minor groove binder.^40^ In addition to maintaining the morphology of chondrocytes, enhancing chondrocyte proliferation and promoting the migration of chondrocytes, TFNA, as a novel DNA material, can exhibit anti-inflammation and anti-oxidation activity in macrophages by suppressing the phosphorylation of members of the MAPK subfamilies.^21,22,41^ Therefore, in this study, we aimed to explore how the combination of TFNA with wogonin affected inflammatory chondrocytes induced by IL-1β and inflamed knee joints in rats.

## Results

### Preparation and characterization of TFNA, wogonin, and, TFNA/wogonin complexes (TWC)

**Fig. 1:**
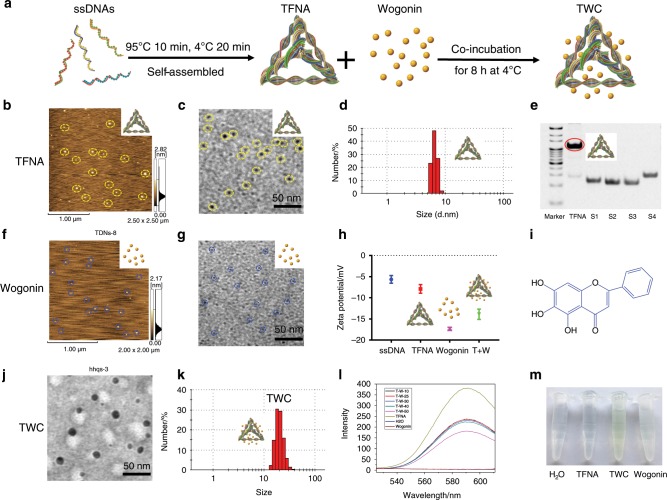
Characterization of TFNA, wogonin and TWC. **a** Schematic of the synthesis of TFNA and the preparation of TWC. **b**, **f** TFNA and wogonin were characterized by AFM. **d**, **k** Analysis of the hydrodynamic size of TFNA and TWC via DLS. **e** PAGE was used to confirm the successful synthesis of TFNA. **h** Zeta potentials of ssDNA, TFNA, wogonin, and TWC were determined. **i** Molecular structure of wogonin. **c**, **g**, **j** TEM was used to verify wogonin, TFNA, and TWC, respectively. **l** Fluorescence emission spectra of a mixture of Gel-Red, λ (Ex)-312 nm, and Gel-Red-TFNA (250 nmol·L^−1^) in the presence of increasing concentrations of wogonin (10–50 µmol·L^−1^) in ddH_2_O. The entrapment efficiency was calculated according to the fluorescence intensity at 600 nm (TWC, TFNA/wogonin complexes). **m** Images of H_2_O, TFNA, TWC, and wogonin

The four specifically designed 63-base-long ssDNA strands (Table 1, strands 1–4) could easily assemble into TFNA through a simple annealing process (Fig. 1a). The successfully synthesized TFNA was characterized by atomic force microscopy (AFM), which is a TFNA verification method that revealed that the height of TFNA in a dry state was ~3 nm (Fig. 1b); this result aligns with that of previous studies.^21,42,43^ Transmission electron microscopy (TEM) was also used to verify TFNA, the size of which was estimated to be <10 nm (Fig. 1c). Dynamic light scattering (DLS) was employed to characterize the synthesized TFNA, and it indicated that the hydrodynamic size of TFNA was ~7 nm (Fig. 1d). As shown in Fig. 1e, the results of polyacrylamide gel electrophoresis (PAGE) demonstrated that the successfully formed TFNA and four ssDNAs were separated based on their mobility; TFNA was observed to move more slowly than the four ssDNAs. In addition, we found that ~90% of the TFNA was effectively formed and displayed a distinct band with a red circle. Taken together, the results indicated that TFNA was efficiently and successfully assembled.

**Table 1:** The sequences of the four designed specific ssDNAs

DNA	Sequence
S1	5′-ATTTATCACCCGCCATAGTAGACGTATCACCAGGCAGTTGAGACGAACATTCCTAAGTCTGAA-3′;
S2	5′-ACATGCGAGGGTCCAATACCGACGATTACAGCTTGCTACACGATTCAGACTTAGGAATGTTCG-3′;
S3	5′-ACTACTATGGCGGGTGATAAAACGTGTAGCAAGCTGTAATCGACGGGAAGAGCATGCCCATCC-3′;
S4	5′-ACGGTATTGGACCCTCGCATGACTCAACTGCCTGGTGATACGAGGATGGGCATGCTCTTCCCG-3′;
Cy5-S1	5′Cy5-ATTTATCACCCGCCATAGTAGACGTATCACCAGGCAGTTGAGACGAACATTCCTAAGTCTGAA-3′.

Based on AFM, the height of dry wogonin was ~1.5 nm (Fig. 1f). By TEM, the size of wogonin was shown to be <5 m (Fig. 1g); as shown in Fig. 1h, the zeta potentials of ssDNA, TFNA, wogonin, and TWC were approximately −8, −6, −17, and −15, respectively. The chemical structure of wogonin is shown in Fig. 1i. Subsequent analyses using TEM and DLS were performed to determine the characteristics of TWC (TFNA loaded with wogonin). Based on the TEM experiments, the size of TWC was ~20 nm. TWC appeared to have formed well-dispersed particles with a uniform circular size (Fig. 1j). Similarly, the size of TWC, as measured by DLS, was ~20 nm (Fig. 1k). A fluorescence spectrophotometer was used to further identify and confirm the successful formation of TWC (Fig. 1l). The complexes of TFNA (250 nmol·L^−1^)-wogonin (50 µmol·L^−1^) had the highest entrapment efficiency of 53.94% ± 15%. From Fig. 1m, it is evident that TWC possesses a more distinct yellow color than pure wogonin. Altogether, these findings indicated that wogonin was successfully loaded into TFNA.

### Uptake of TFNA and ssDNA by normal and inflammatory chondrocytes

**Fig. 2:**
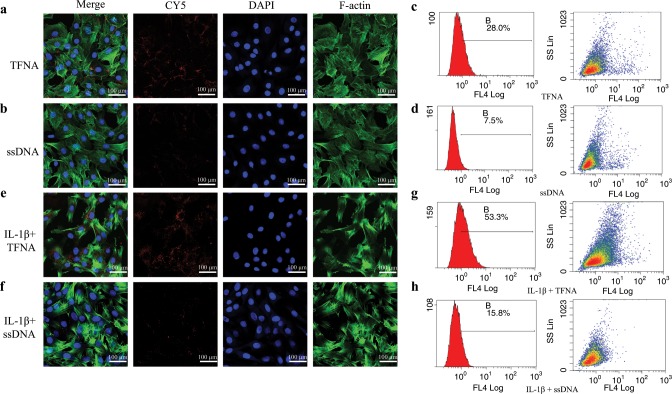
The ability of ssDNA and TFNA to enter normal and inflammatory chondrocytes was detected by immunofluorescence and flow cytometry. **a**, **b**, **e**, **f** ssDNA-CY5 and TFNA-CY5 were internalized by normal and inflammatory chondrocytes, respectively (CY5, red; cytoskeleton, green; nucleus, blue) (scale bars: 100 µm). **c**, **d**, **g**, **h** Cellular uptake (by normal and inflammatory chondrocytes) of ssDNA-CY5 and TFNA-CY5 was detected by flow cytometry

We detected the capability of TFNA and ssDNA to enter chondrocytes in normal and inflammatory states using immunofluorescence and flow cytometry (Fig. 2). TFNA and ssDNA were labeled with CY5 (red) dye, which can be used to visualize these materials. Large amounts of TFNA were observed to have been internalized into normal chondrocytes; however, faint fluorescence was detected from ssDNA in these cells (Fig. 2a, b). According to the results of flow cytometry (Fig. 2c, d), normal chondrocytes were also observed to absorb more TFNA than ssDNA following incubation with the DNA materials for 6 h. Similar to normal chondrocytes, TFNA was easily and effectively delivered into inflammatory chondrocytes, but ssDNA could hardly enter these cells (Fig. 2e, f). By flow cytometry, the cellular uptake level of TFNA was shown to be significant (~53.3%). In contrast, only 15.8% of ssDNAs were internalized into these cells (Fig. 2g, h), as observed by immunofluorescence.

### The expression of various genes related to OA before and after treatment

**Fig. 3:**
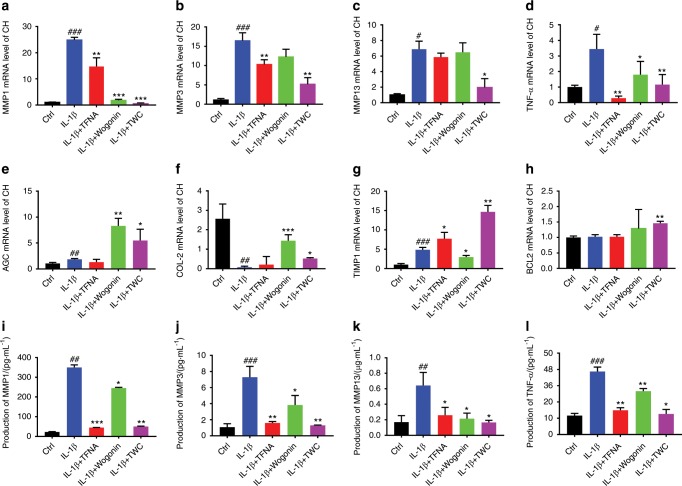
qPCR and ELISA analysis the expressions of MMPs, inflammatory factors and cartilage related genes in vitro. qPCR analysis of the expression of MMPs [MMP1 (**a**), MMP3 (**b**), MMP13 (**c**)], inflammatory factors (*TNF-α*) (**d**), chondrogenic markers (*AGC* (**e**), *COL-II* (**f**)), *TIMP1*(**g**), and *BCL2* (**h**) in chondrocytes treated with IL-1β and various materials. The gene expression was normalized to that of the housekeeping gene *GAPDH*. The secreted levels of MMPs [MMP1 (**i**), MMP3 (**j**), MMP13 (**k**)], and TNF-α (**l**) in the supernatants of normal chondrocytes and inflammatory chondrocytes treated with various materials, as assayed by ELISA. Statistical analysis: (^*^) compared to the IL-1β group; ^*^P < 0.05, ^**^P < 0.01, ^***^P < 0.001. (^#^) Compared to the control group; ^#^*P* < 0.05, ^##^*P* < 0.01, ^###^*P* < 0.001

The genes relevant to OA were detected by real-time qPCR. As shown in Fig. 3a–d, the gene expression of *MMPs* (*MMP1*, *MMP3*, and *MMP13*) and *TNF-α* were downregulated after treatment with TFNA, wogonin, and TWC. In addition, IL-1β led to remarkable inflammation, as the gene expression of the *MMPs* and *TNF-α* was notably enhanced following the addition of IL-1β. Compared to IL-1β, TWC was observed to significantly inhibit the gene expression of *MMPs* and *TNF-α* (*MMP1*, 0.02-fold; *MMP3*, 0.375-fold; *MMP13*, 0.29-fold; *TNF-α*, 0.37-fold). Moreover, compared to that in the IL-1β groups, the gene expression of *AGC* and collagen-II (*COL-*II*)* were upregulated in inflammatory chondrocytes after treatment with TWC (*AGC*, 5-fold; *COL-*II, 3-fold) (Fig. 3e, f). Similarly, the mRNA expression of tissue inhibitor of metalloproteinase 1 (*TIMP1)* in the TWC group was higher than that observed in the IL-1β group (*TIMP1*, 2.22-fold) (Fig. 3g). For B-cell lymphoma 2 (*BCL2)*, a higher gene expression level was observed in the TWC group than in the other groups (*BCL2*, 1.5-fold compared to the IL-1β groups) (Fig. 3h).

### Enzyme-linked immunosorbent assay (ELISA) of inflammatory factors and MMPs in vitro and in vivo and the expression of NF-κB p65 and IκBα as detected by WB

**Fig. 4:**
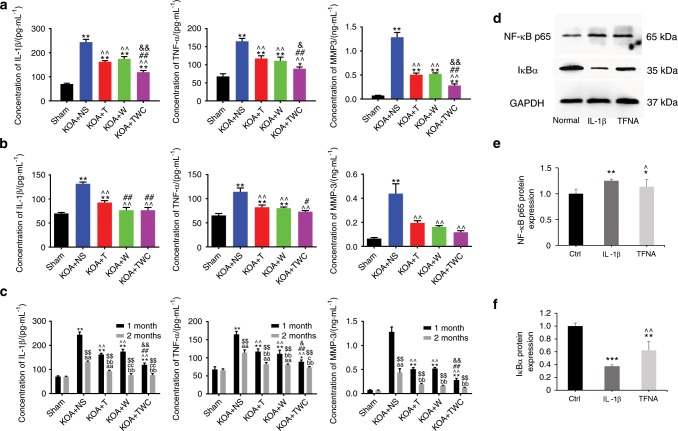
ELISA analysis of the protein expressions of inflammatory factors and MMPs in vivo and the expression of NF-κB p65 and IκBα as detected by western blot. **a**, **b** ELISA results of IL-1β, TNF-α, and MMP3 protein expression in joint fluid from rats in each group at 1 and 2 months. Statistical analysis: (^*^) compared to the sham group; ^*^P < 0.05, ^**^P < 0.01. (^^^) Compared to the KOA + NS group; ^^^^*P* < 0.01. (^#^) Compared to the KOA + T group; ^#^*P* < 0.05, ^##^*P* < 0.01. (^&^) Compared to the KOA + wogonin group; ^&^*P* < 0.05, ^&&^*P* < 0.01. **c** Comparison of the 1-month and 2-month data for the protein expression of IL-1β, TNF-α, and MMP3. Statistical analysis: (*) compared to the sham group (1 month); *P < 0.05, ^**^P < 0.01. (^^^) Compared to the KOA + NS group (1 month); ^^^^*P* < 0.01. (^#^) Compared to the KOA + T group (1 month); ^##^*P* < 0.01. (^&^) Compared to the KOA + wogonin group (1 month); ^&^*P* < 0.05, ^&&^*P* < 0.01. (^a^) Compared to the sham group (2 months); ^aa^*P* < 0.01. (^b^) Compared to the KOA + NS group (2 months); ^bb^*P* < 0.01. (^c^) Compared to the KOA + T group (2 months); ^c^*P* < 0.05, ^cc^*p* < 0.01. (^$^) Compared to the KOA + wogonin group (2 months); ^$$^*P* < 0.01. (T, TFNA; W, wogonin.) **d** Protein expression of NF-κB p65 and IκBα was analyzed by western blot analysis. **e**, **f** Quantification of NF-κB p65 and IκBα expression using a *t* test. Statistical analysis: (^*^) compared to the control group; ^*^P < 0.05, ^**^P < 0.01, ^***^P < 0.001. (^^^) Compared to the IL-1β group; ^^^*P* < 0.05, ^^^^*P* < 0.01

The secreted levels of MMPs (MMP1, MMP3, and MMP13) and TNF-α in the culture supernatants of treated OA chondrocytes were measured using ELISA (Fig. 3i–l). As shown in Fig. 3i, j, TWC effectively suppressed the expression of MMP1 and MMP3 after the induction of an inflammatory reaction by IL-1β. The secreted level of MMP13 in the TWC group was also observed to be lower than that in the IL-1β group (Fig. 3k), and the TNF-α expression level was downregulated after treatment with TWC (Fig. 3l). The secreted levels of IL-1β, TNF-α, and MMP3 in the knee joint fluid of rats were examined by ELISA (Fig. 4a–c). Compared to those in the normal saline (knee osteoarthritis (KOA) + NS) group, the expression levels were upregulated after modeling (KOA) and downregulated after treatment with TFNA, wogonin, and TWC at 1 month and 2 months, respectively (Fig. 4a, b). As shown in Fig. 4c, these three factors might be further inhibited after treatment with these materials at 2 months compared to 1 month. The activation of the nuclear factor-kappa B (NF-κB) pathway, which is activated by IL-1β, was detected using western blot mainly by assaying the protein expression of NF-κB p65 and IκBα (Fig. 4d–f). As expected, TFNA significantly suppressed the activation of NF-κB p65 and inhibited the degradation of IκBα, both of which were induced by IL-1β in chondrocytes.

### Results of micro-CT after 1 and 2 months

**Fig. 5:**
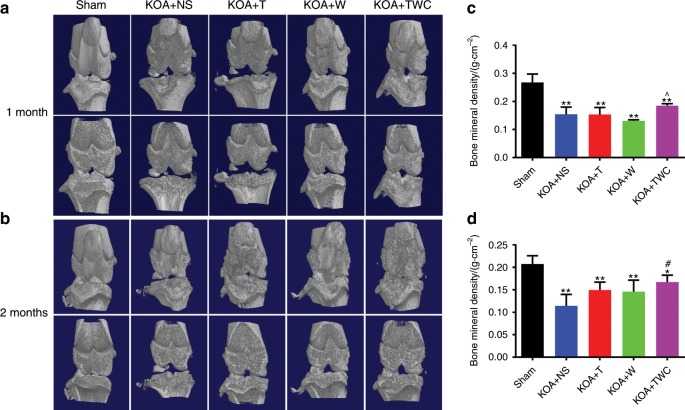
Micro-CT images of rat knee joints. **a**, **b** Micro-CT analysis was used to evaluate the knee joints of rats after treatment with different materials at 1 month and 2 months, respectively. **c**, **d** The BMD was calculated by phantom scanning of the results of micro-CT at 1 month and 2 months, respectively. Statistical analysis: (^*^) compared to the sham group; ^*^P < 0.05, ^**^P < 0.01. (^#^) Compared to the KOA + NS group; ^#^*P* < 0.05. (^^^) Compared to the KOA + wogonin group; ^^^*P* < 0.05

The bone mineral density (BMD) of regenerated tissues in the rat knee joint was examined by micro-CT at 1 month and then at 2 months. Based on this analysis, the BMD was measured to further estimate the degree of bone healing in the rat knee joint following treatment with TFNA, wogonin, and TWC. Representative images of 3D micro-CT are shown in Fig. 5a (1 month) and Fig. 5b (2 months). At 1 month, there was a slight increase in the BMD for the TWC group (Fig. 5c), and at 2 months, this value was increased for the KOA + TFNA, KOA + wogonin, and KOA + TWC groups (Fig. 5d). In addition, the BMD in the KOA + TWC group was dramatically enhanced when compared to that in the KOA + NS group.

### Hematoxylin and eosin (H&E), Masson, and Safranin-O staining

**Fig. 6:**
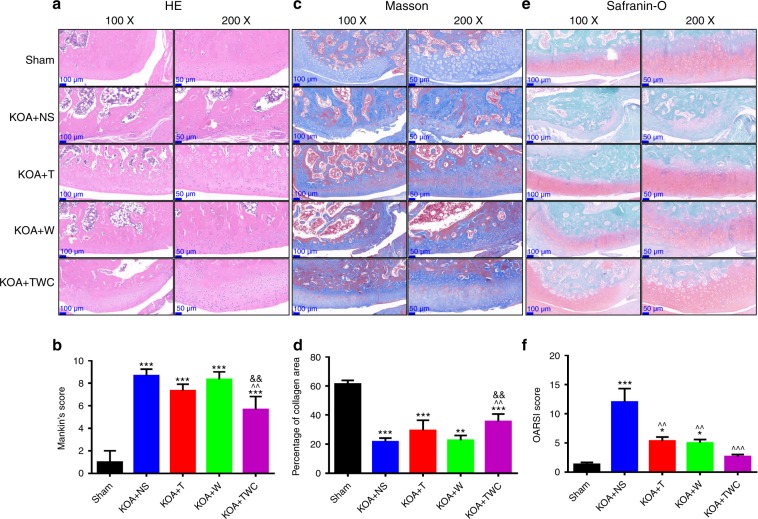
Images of rat cartilage sections at 1 month when stained by H&E, Masson, and Safranin-O. **a** Macroscopic images of H&E staining, **b** Mankin’s score for articular cartilage from all groups, **c** macroscopic images of Masson staining, **d** collagen area (%) of articular cartilage from all groups, **e** macroscopic images of Safranin-O staining, **f** OARSI semiquantitative scores of articular cartilage for all groups. Statistical analysis: (^*^) compared to the sham group; ^*^P < 0.05, ^**^P < 0.01, ^***^P < 0.001. (^^^) Compared to the KOA + NS group; ^^^^*P* < 0.01, ^^^^^*P* < 0.001. (^&^) Compared to the KOA + wogonin group; ^&&^*P* < 0.01

**Fig. 7:**
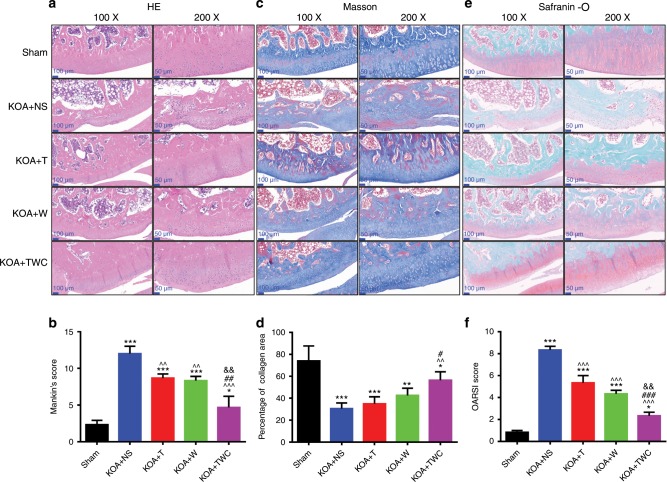
Images of rat cartilage sections at 2 months following H&E, Masson, and Safranin-O staining. **a** Macroscopic images of H&E staining, **b** The Mankin’s score for articular cartilage from all groups, **c** macroscopic images of Masson staining, **d** collagen area (%) of articular cartilage from all groups, **e** macroscopic images of Safranin-O staining, **f** OARSI semiquantitative scores of articular cartilage for all groups. Statistical analysis: (^*^) compared to the sham group; ^*^P < 0.05, ^**^P < 0.01, ^***^P < 0.001. (^^^) Compared to the KOA + NS group; ^^^^*P* < 0.01, ^^^^^*P* < 0.001. (^#^) Compared to the KOA + T group; ^#^*P* < 0.05, ^##^*P* < 0.01, ^###^*P* < 0.001. (^&^) Compared to the KOA + wogonin group; ^&&^*P* < 0.01

Representative images of H&E, Masson, and Safranin-O staining at 1 and 2 months are shown in Figs. 6 and 7, respectively. The H&E results for the knee joints of rats in each group are shown in Figs. 6a, b (1 month) and 7a, b (2 months). Although bone damage was observed in the KOA + NS group, in the other KOA model groups, namely, the KOA + TFNA, KOA + wogonin and KOA + TWC groups, intact articular cartilage, a smooth cartilage surface, and an orderly arrangement were observed. In addition, the cartilage layer in the KOA + TFNA, KOA + wogonin, and KOA + TWC groups was much thicker than that in the KOA + NS group, thereby indicating that TFNA, wogonin, and TWC administration could alleviate bone damage. As shown in Figs. 6c, d (1 month) and 7c, d (2 months), following Masson staining, the cartilage collagen was blue, while the calcified cartilage and bone trabecular collagen were red. These results indicate the severe loss of cartilage collagen in the KOA model. After treatment with TFNA, wogonin, and TWC, however, these groups experienced the specific improvement of cartilage collagen loss. TFNA, wogonin, and TWC were also observed to slow the rate of cartilage collagen loss. Similarly, in Figs. 6e, f (1 month) and 7e, f (2 months), Safranin-O staining showed that the structure of the articular cartilage in the KOA + TFNA, KOA + wogonin, and KOA + TWC groups was more intact than that found in the KOA + NS group. The cartilage surface in the three treatment groups was smoother than that in the KOA + NS group, and although the cartilage matrix was unevenly distributed to a small extent, the chondrocytes proliferated and were arranged neatly. Treatment with TFNA, wogonin, and TWC ameliorated bone injury, and TWC produced better effects than the other test materials.

### Transferase dUTP nick-end labeling (TUNEL) staining

**Fig. 8:**
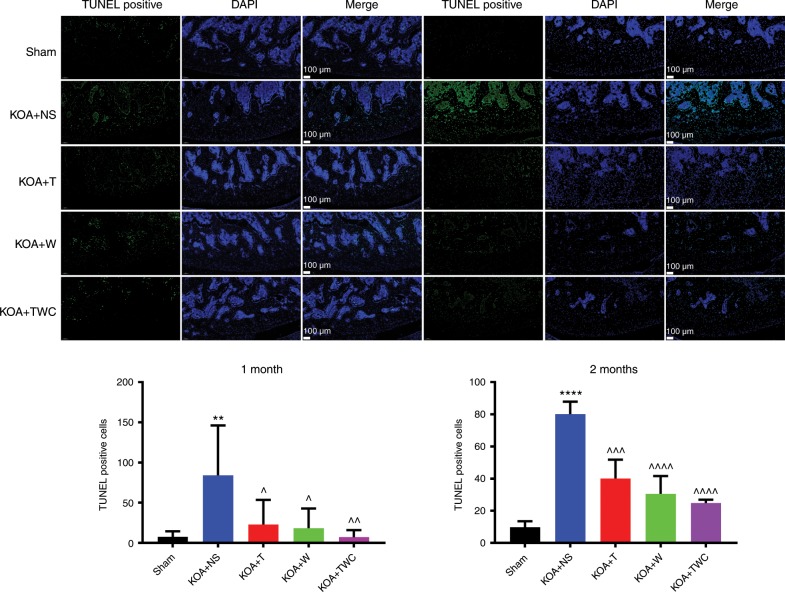
TUNEL staining of joint cartilage from different groups at 1 month and 2 months following treatment with the different materials. Green indicates TUNEL-positive cells. Statistical analysis: (^*^) compared to the sham group; ^**^P < 0.01, ^****^P < 0.0001. (^^^) compared to the KOA + NS group; ^^^*P* < 0.05, ^^^^*P* < 0.01, ^^^^^*P* < 0.001, ^^^^^^*P* < 0.0001

According to the results of the TUNEL staining performed at 1 and 2 months, almost no apoptosis was found on the surface and in the external and middle layers of articular cartilage in the sham group. However, apoptosis of articular cartilage cells in the KOA + NS group was significantly increased (Fig. 8). Compared to that in the KOA + NS group, apoptosis of the articular cartilage cells in the KOA + TFNA, KOA + wogonin, and KOA + TWC groups was decreased by varying degrees. Among these groups, the apoptosis of chondrocytes was the lowest in the KOA + TWC group. Therefore, TFNA, wogonin, and especially TWC inhibited the apoptosis of chondrocytes in the articular cartilage.

### Immunofluorescence of COL-II and AGC

**Fig. 9:**
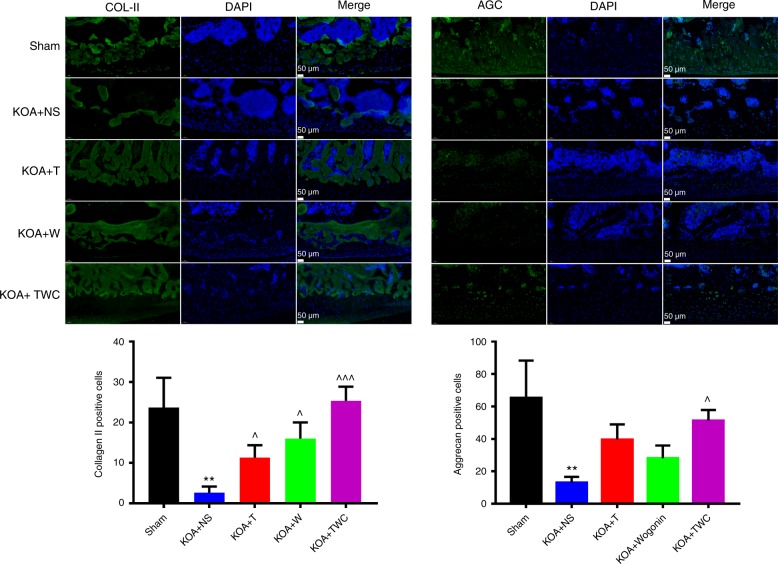
Immunofluorescence analysis and quantification of COL-II and AGC at 1 month. Statistical analysis: (^*^) compared to the sham group; ^**^P < 0.01. (^^^) Compared to the KOA + NS group; ^^^*P* < 0.05, ^^^^^*P* < 0.001

**Fig. 10:**
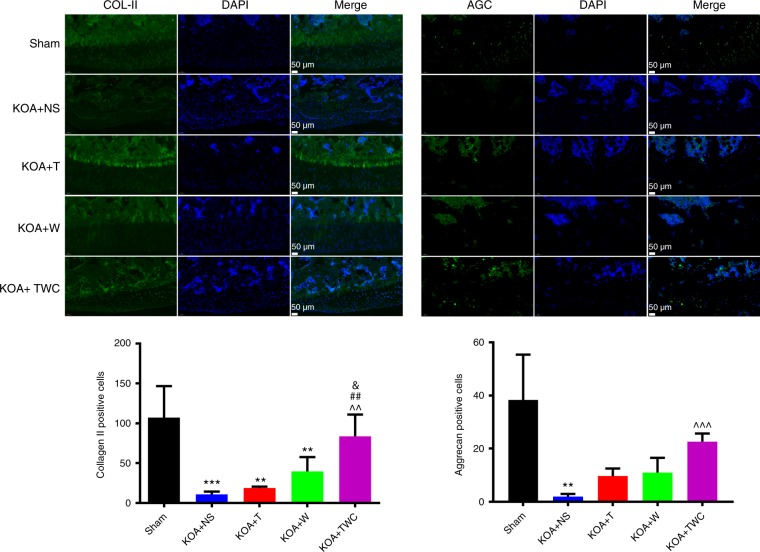
Immunofluorescence analysis and quantification of COL-II and AGC at 2 months. Statistical analysis: (^*^) compared to the sham group; ^**^P < 0.01, ^***^P < 0.001. (^^^) Compared to the KOA + NS group; ^^^^*P* < 0.01, ^^^^^*P* < 0.001. (^#^) compared to the KOA + T group; ^##^*P* < 0.01. (^&^) compared to the KOA + Wogonin group; ^&^*P* < 0.05

Based on the immunofluorescence analysis, COL-II and AGC expression were markedly decreased in the KOA + NS group both at 1 and 2 months (Figs. 9 and 10). After treatment with TFNA, wogonin, and TWC, the COL-II and AGC expression levels were increased. Based on the results at both 1 and 2 months and compared to those of the other materials, TWC exerted the greatest effect in promoting the expression of the two chondrogenic markers (Figs. 9 and 10). Taken together, these results demonstrate that TWC can protect cartilage tissue by enhancing the expression of chondrogenic markers.

## Discussion

TFNA, a novel and promising DNA nanomaterial possessing excellent structural stability, high mechanical strength, and modification versatility, has been widely applied in various fields of biomedicine.^27–30^ Compared to various nanochemical polymers (upconversion nanotransducer-based nanocomplexes, nanocomposite hydrogels, or supramolecular hydrogels), TFNA not only possesses good biocompatibility and biodegradability and the ability to permeate cells but can also be functionalized via modification with DNA fragments, RNAs, polypeptide monomers, and small-molecule drugs.^31–33,44–46^ In our previous studies, we found that TFNA can enhance the proliferation and migration of chondrocytes and maintain their morphology at an optimum concentration of 250 nmol·L^−1^.^21,22^ Upon consideration of these characteristics, TFNA was used in combination with wogonin, a naturally occurring flavonoid with various biological properties, such as anti-inflammatory and anti-cancer activity.^34,35^ In our study, we innovatively applied these two materials to the treatment of inflammatory chondrocytes induced by IL-1β and inflamed knee joints in rats.

TFNA was successfully self-assembled from four specially designed ssDNAs, as observed by AFM, DLS, PAGE, TEM, and zeta potential analyses.^21,22,24,27–33^ Subsequently, wogonin was loaded into TFNA (TWC) at the optimum concentration. Based on the results from TEM, DLS, and fluorescence spectrophotometry, TWC was shown to have been efficiently and effectively formed. We then proceeded to use TFNA, wogonin, and TWC to treat inflammatory chondrocytes and OA. We found that all three materials enhanced chondrocyte regeneration and inhibited inflammation. However, when compared to TFNA and wogonin, TWC exhibited the best therapeutic effect. TFNA can be efficiently internalized by mammalian cells, which is essential for effective intracellular drug delivery and subsequent treatment.^21,22,24,27,28,30,47^ By using immunofluorescence and flow cytometry in the present study, we found that large amounts of TFNA could easily enter normal chondrocytes and inflammatory chondrocytes, while ssDNAs could not. Moreover, the absorption of TFNA by inflammatory chondrocytes was greater than that by normal chondrocytes, which is indeed important for subsequent therapy.

In our study, TFNA, wogonin, and TWC demonstrated the potential to inhibit inflammation and promote chondrocyte regeneration in vitro and in vivo. By qPCR and ELISA, we found that TWC (i.e., 250 nmol·L^−1^ TFNA and 50 μmol·L^−1^ wogonin) can downregulate the expression of MMPs (MMP1, MMP3, and MMP13) and TNF-α, which play critical roles in maintaining the balance between synthesis and degradation in normal cartilage extracellular matrix (ECM). It was also suggested that the expression of MMPs was markedly elevated, while the mRNA expression levels of anabolic factors (COL-II and AGC) were significantly downregulated in chondrocytes from individuals suffering from OA.^48–50^ In OA, three MMPs (MMP1, MMP3, and MMP13), especially MMP13, exert a primary function and play an important role in chondrocyte-mediated cartilage matrix degeneration.^49,51^ In our study, the expression of MMPs (MMP1, MMP3, and MMP13) and TNF-α were decreased in the TFNA, wogonin, and TWC groups. TNF-α and IL-1β are important inflammatory mediators driving the expression of catabolic enzymes.^52^ Both COL-II and AGC are major critical ECM components in the articular cartilage, and they can be degraded by MMPs, especially MMP13, in OA.^49^ We found that the mRNA expression levels of *TIMP1*, *BCL2*, *AGC*, and *COL-*II were upregulated after treatment with TFNA, wogonin, and TWC in inflammatory chondrocytes. TIMPs, such as TIMP1, are natural inhibitory proteins of MMPs. Previous data demonstrated that TIMP1 is a functional regulator of MMP13-related chondrocyte senescence and changes that occur during aging in the cartilage matrix.^53,54^ BCL2, an anti-apoptotic factor, can notably inhibit the apoptosis of chondrocytes when its expression is increased.^55,56^ Our results showed that TWC inhibits MMPs (*MMP1*, *MMP3*, *MMP13*) and *TNF-α* and upregulates anabolic gene (*COL-*II and *AGC*), *TIMP1*, and *BCL2* expression in IL-1β-treated chondrocytes.

Based on the results of qPCR and ELISA, we selected TFNA (250 nmol·L^−1^), wogonin (50 µmol·L^−1^), and TWC (TFNA, 250 nmol·L^−1^; wogonin, 50 µmol·L^−1^) for the animal experiments. According to the ELISA results in vivo, the expression of IL-1β, TNF-α, and MMP3 were significantly decreased after treatment with TFNA, wogonin, and TWC at both 1 and 2 months. Of the materials used in this study, TWC exhibited the most evident inhibitory effect on inflammation. Previously, it was suggested that IL-1β and TNF-α play crucial roles in the progression of OA and are strongly related to functional changes in the articular cartilage.^57^ Not only can IL-1β induce the expression of inhibiting factors such as NF-κB family members, but it can also enhance the synthesis of MMPs in chondrocytes and synoviocytes.^58^ Likewise, we detected and found that TFNA significantly suppressed the activation of NF-κB p65 and inhibited the degradation of IκBα, both of which are induced by IL-1β in chondrocytes. Micro-CT, which was employed to examine bone density, revealed that the BMD of regenerated tissues was higher in the KOA + TFNA, KOA + Wogonin, and especially the KOA + TWC groups than in the KOA + NS group. These materials were shown to accelerate bone formation following OA. H&E, Masson, and Safranin-O staining are commonly used to evaluate tissue morphology and the interface between native and newly formed tissues.^59–61^ Histological examinations of knee joint sections revealed the superior effects of TWC compared to those of free TFNA and wogonin. This was indicated by the smoother articular cartilage surface that was observed when knee joints were treated with TWC versus the other materials. This also suggested that chondrocyte proliferation was promoted, and the arrangement of chondrocytes was more orderly in the treated groups, particularly the TWC group. As indicated by TUNEL staining, a decreased number of apoptotic cells were found in the treatment group compared to that in the KOA + NS group, which was aligned with the results of the histological staining. In the evaluation of the therapeutic efficacy, the expression of COL-II and AGC, which are ECM macromolecules reflecting cartilage ECM degradation, were tested by immunofluorescence.^62,63^ The expression of COL-II and AGC were significantly upregulated in the treatment groups, especially the KOA + TWC group, compared to the KOA + NS group, indicating the suppression of the progression of OA in the knee.

## Conclusion

**Fig. 11:**
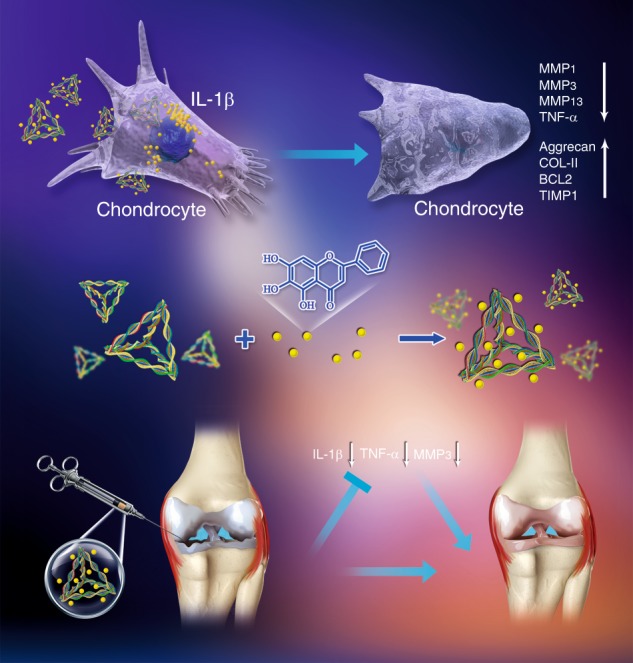
Schematic diagram illustrating the anti-inflammatory and chondroprotective effects of TWC in cell and rat models of OA. The middle section shows the process of TWC synthesis. The upper section shows the TWC-mediated attenuation of chondrocyte inflammation in the OA cell model. The lower section shows the TWC-mediated alleviation of OA in the rat model

In this study, we successfully synthesized a novel substance, TWC, which was applied to treat OA (Fig. 11). Large amounts of TFNA were demonstrated to be quickly and easily internalized into inflammatory chondrocytes. TFNA, wogonin, and TWC also showed potential to effectively alleviate inflammatory reactions in vitro and in vivo and to prevent the destruction of rat cartilage; however, the effects of TWC were superior to those of the other two. Based on our results, these three materials can notably inhibit cell apoptosis, increase chondrogenic marker (*COL-*II and *AGC*) expression, and suppress the expression of inflammatory mediators (IL-1β and TNF-α) in a rat knee model of OA. As TWC was better at delaying the progression of OA than the other two materials, we believe that TWC could be a potential injectable form of therapy for OA.

## Materials and methods

### Materials

The ssDNA strands (Table 1) that were designed with our sequences were synthesized by Takara (Otsu, Japan). Fetal bovine serum (FBS), penicillin–streptomycin solution, phosphate-buffered saline (PBS), 0.25% (w/v) trypsin-ethylenediaminetetraacetic acid solution, type II collagenase, and Dulbecco’s modified Eagle medium (DMEM) were obtained from GE Healthcare (Little Chalfont, UK). Dimethyl sulfoxide (DMSO) was purchased from MP Biomedicals (California, USA). Wogonin was obtained from Coolaber (China, Beijing). Tris-HCl, MgCl_2_, bicinchoninic acid (BCA), and sodium dodecyl sulfate (SDS) were acquired from Bio-Rad (Hercules, CA). The culture vessels and culture plates were procured from Corning (NY, USA). Polyvinylidene difluoride (PVDF) membranes were acquired from Millipore (MA, USA). Antibodies (COL-II and AGC) were purchased from Abcam (Cambridge, UK). Phalloidin and DAPI were obtained from Cytoskeleton (Denver, USA). The 4% paraformaldehyde solution was acquired from Solarbio (Beijing, China). The SYBR® Green I polymerase chain reaction (PCR) master mix, RNeasy® Plus Mini Kit, and DNase I were obtained from Takara (Tokyo, Japan).

### Cell culture

Sprague-Dawley (SD) rats were acquired under strict accordance with the governing ethical principles. The protocol used in the study was reviewed and approved by our Institutional Review Board (IRB). Newborn SD rats were used to generate the articular chondrocytes. Briefly, cartilage tissues were cut into pieces, treated with 0.25% trypsin for 30 min, and coincubated with type II collagenase at 37 °C for 2.5 h; washing with PBS was performed after each step. After centrifugation at 220 × g for 8 min at room temperature, chondrocytes were collected and resuspended in DMEM with 10% FBS. Subsequently, the chondrocytes were seeded in T25 culture vessels and cultured in an incubator.

### OA cell models and rat models

Rat chondrocytes from passages 2 to 4 were used in this study. Cells were incubated with the different materials (TFNA, wogonin, or TWC) for 2 h and then treated with IL-1β (10 ng·mL^−1^) for 24 h. The animal experiments were carried out according to the guidelines of the animal ethics committee of Sichuan University. Male Wistar rats (weight, 190 g ± 20 g) were anesthetized, and then the right knee joint was shaved to clean and sterilize the area. After separating the skin and muscle, we first removed the exposed anterior and posterior cruciate ligaments and then removed the medial meniscus. The wound was smeared with chloramphenicol eye ointment to prevent infection after hemostasis and then cleaned and sutured. Rats with OA were injected with normal saline and the different compounds (TFNA, wogonin, or TWC) in the knee joint cavity (100 μL per time per day). Rats were examined after 4 and 8 weeks of continuous treatment administration.

### Preparation and characteristics of TWC

As reported previously,^42^ four ssDNA strands were added to TM buffer (10 mmol·L^−1^ Tris-HCl and 50 mmol·L^−1^ MgCl_2_, pH 8.0) and then amplified using a PCR system (95 °C ~ 10 min, 4 °C ~ 20 min). The successful formation of TFNA was confirmed by 8% PAGE. Wogonin was dissolved in DMSO and diluted with PBS. TWC was synthesized by adding wogonin solution to the TFNA solution, and the mixture was incubated for 8 h at 4 °C on a shaker. The surface structures and properties of TFNA and wogonin were characterized by AFM using an SPM-9700 instrument (Shimadzu, Kyoto, Japan). Images of TFNA, wogonin, and TWC were acquired using a TEM at 70 kV (HT770, Hitachi, Japan). DLS was performed using a ZetaPALS analyzer (Brookhaven Instruments, Holtsville, NY, USA). A Zetasizer Nano ZS90 (Malvern Instruments Ltd., UK) was used to detect the zeta potential of ssDNA, TFNA, wogonin, and TWC. Fluorescence spectrophotometry (Shimadzu RF-5301PC, Japan) was performed to determine the entrapment efficiencies of TWC.

### Cellular uptake of TFNA

Normal chondrocytes and inflammatory chondrocytes were seeded in culture plates and incubated for 24 h. After washing with PBS, they were coincubated with Cy5-ssDNA (250 nmol·L^−1^) and Cy5-TFNA (250 nmol·L^−1^) in DMEM with 1% FBS for 8 h. After washing with PBS, the cells were fixed with 4% (w/v) paraformaldehyde. FITC-labeled phalloidin (FITC: PBS = 7:1 000) and DAPI (DAPI: PBS = 1:1 000) were used to stain the cytoskeleton and nuclei, respectively. Meanwhile, a flow cytometer (FC500 Beckman, IL, USA) was used to detect the uptake of ssDNA and TFNA by chondrocytes in different states (normal and inflammatory).

### Quantitative real-time PCR (qPCR)

An RNeasy® Plus Mini Kit was used to extract the total RNA from chondrocytes, and a spectrophotometer was used to quantify the RNA samples. After quantification, each total RNA sample (~0.5 µg) was reverse-transcribed by using cDNA synthesis kits. Target mRNA expression was assessed by qPCR with SYBR® Green I PCR master mix and an ABI 7300 thermal cycler (Applied Biosystems, Foster City, CA, USA). All primers (the working concentration of each primer was 0.4 μmol·L^−1^) in Table 2 were designed using BLAST searches, and *GAPDH* amplification was used as the control. The cycling conditions for the qPCR procedure consisted of denaturation for 3 min at 94 °C followed by 40 cycles of 5 s at 94 °C and 34 s at 60 °C.

**Table 2:** The primer sequences of the housekeeper genes and related genes designed for qPCR

mRNA	Product length	Primer pairs	
GAPDH	233 bp	Forward	ACAGCAACAGGGTGGTGGAC
		Reverse	TTTGAGGGTGCAGCGAACTT
COL-II	116 bp	Forward	TCAAGTCGCTGAACAACCAG
		Reverse	G TCTCCGCTCTTCCACTCTG
Aggrecan	137 bp	Forward	GCAGCACAGACACTTCAGGA
		Reverse	CCCACTTTCTACAGGCAAGC
MMP1	136 bp	Forward	GCTTAGCCTTCCTTTGCTGTTGC
		Reverse	GACGTCTTCACCCAAGTTGTAGTAG
MMP3	77 bp	Forward	CTGGGCTATCCGAGGTCATG
		Reverse	TGGACGGTTTCAGGGAGGC
MMP13	131 bp	Forward	CCCAGATGATGACGTTCA AGGA
		Reverse	CTCGGAGACTAGTAATGGCATCAAG
TIMP1	96 bp	Forward	TCCCTGTTCAGCCATCCCTTG
		Reverse	TCGCTCTGGTAGCCCTTCTC
BCL-2	214 bp	Forward	ATACCTGGGCCACAAGTGAG
		Reverse	TGATTTGACCATTTGCCTGA
TNF-α	125 bp	Forward	AGAAAAGCAAGCAACCAGCC
		Reverse	TCTGCCAGTTCCACATCTCG

### Western blot analysis

Chondrocytes were washed, harvested, and lysed in lysis buffer. A BCA assay was then conducted to determine the protein concentration. Proteins were denatured by boiling in SDS buffer and were separated by SDS-PAGE. After transferring the proteins to PVDF membranes, blocking was performed using skim milk for 30 min. This was followed by a 12-h incubation with the primary antibodies (anti-NF-κB p65, Abcam, ab16502, 1:800; anti-IκBα, Abcam, ab32518, 1:1 000) and a 45-min incubation with the secondary antibodies. Finally, the immunoreactivity was visualized by enhanced chemiluminescence.

### ELISA

MMP-1, -3, and -13 and TNF-α in the supernatants derived from chondrocytes treated with different materials were tested by ELISA. A rat *MMP1* ELISA kit (Cloud-clone Corp. SEA097Ra), *MMP3* ELISA kit (Cloud-clone Corp. SEA101Ra), *MMP13* ELISA kit (Cloud-clone Corp. SEA099Ra), and a rat *TNF-α* ELISA kit (Cloud-clone Corp. SEB133Ra) were used in this study. MMP3, TNF-α, and IL-1β produced in the knee joint fluid of rats treated with TFNA, wogonin, and TWC were detected using an *MMP3* ELISA kit (Cloud-clone Corp. SEA101Ra), a *TNF-α* ELISA kit (Cloud-clone Corp. SEB133Ra), and an *IL-1β* ELISA kit (Cloud-clone Corp. SEA073Ra), respectively, after 1 and 2 months. A microplate reader was then used to detect the optical density at 450 nm. All experiments were carried out in triplicate.

### Microcomputed tomography (Micro-CT)

The total right knee joint in a rat (including the total knee joint, distal femur, and proximal tibia) was removed, which was followed by the removal of the attached muscles and ligaments. After fixing in a 40 g·L^−1^ paraformaldehyde solution, the sections were removed, placed on the workstation stand, and positioned at the scanning center to ensure that the observed sections were within the CT scan range. Each sample was scanned using the Skyscan 1174 Micro-CT Scanner software (voltage, 50 kV; current, 800 μA). The scan was performed at a scan resolution of 14.5 μm and with a field of view of 1 304 × 1 024. A total of 125 consecutive slices of the femoral epiphyseal plate, which included the bone marrow cavity at a thickness of 1.8 mm, were used to image the three-dimensional reconstructed region of interest. The three-dimensional images were reconstructed by N-Recon software, and the BMD was determined using CT-AN software.

### Histological analysis

All knee joints were fixed in 40 g·L^−1^ paraformaldehyde solution for 3 days, decalcified using formalin-ethylenediaminetetraacetate solution for 6 weeks, dehydrated with gradient ethanol solutions, embedded in paraffin blocks, and cut into slices. H&E, Masson, and Safranin-O staining were used to stain the slices. A tissue scanner (Aperio, ScanScope XT, USA) was used to scan all stained slices. Under magnification at ×100 and ×200, the slices were imaged and analyzed using the built-in software (Aperio, Image Scope, USA). The histological analysis of OA was performed using the Osteoarthritis Research Society International (OARSI) semiquantitative scoring scale and a modified Mankin’s score based on histomorphology. Based on Masson staining, the collagen area (%) of the articular cartilage derived from all groups was evaluated.

### TUNEL assay

Apoptosis in the knee joint cartilage was confirmed by TUNEL staining (Roche, Mannheim, Germany). Three sections from each knee in the different groups were used for TUNEL histochemistry. The nuclei of the chondrocytes were counterstained with DAPI. The percentage of TUNEL-positive nuclei among the DAPI-labeled nuclei for each individual was used to identify chondrocyte apoptosis and the average for each treatment group.

### Immunofluorescence

Immunofluorescence was used to examine the localization and expression of COL-II and AGC. All sections were deparaffinized, rehydrated and incubated at 60 °C for 12 h. To quench the endogenous peroxidase activity after incubation with a nonspecific staining blocking reagent, a 15-min incubation was performed with 3% H_2_O_2_. After incubation with the primary antibodies [COL-II (ab34712, Abcam, 1:200) and AGC (ab3773, Abcam, 1:100)], 10% normal goat serum was added, followed by the subsequent incubation with the secondary antibody for 1.5 h. Finally, the nuclei were counterstained with DAPI (DAPI: PBS =1:1 000). All images were acquired using a fluorescence microscope (×200). Image-Pro Plus 6.0 (Media Cybernetics) was used to analyze the results of these images.

### Statistical analysis

The data analysis was performed using SPSS 16.0 statistical software. All data are expressed as the mean ± standard deviation. One-way ANOVA was used to compare three or more groups. The Student’s *t* test was used to compare two groups. A *P* value < 0.05 was considered statistically significant.

## Data Availability

All data included in this study are available from the corresponding author upon request.
